# *Bacillus* spp.: potent microfactories of bacterial IAA

**DOI:** 10.7717/peerj.7258

**Published:** 2019-07-23

**Authors:** Shabana Wagi, Ambreen Ahmed

**Affiliations:** Department of Botany, University of the Punjab, Lahore, Punjab, Pakistan

**Keywords:** IAA, *Bacillus*, TLC, FTIR, Phosphate solubilization

## Abstract

**Background:**

Auxin production by bacteria is one of the most important direct mechanisms utilized by plant growth-promoting bacteria (PGPB) for the betterment of plants naturally because auxin is a plant friendly secondary metabolite synthesized naturally by bacteria, and hence improves the growth of associated plants. So, the current study focuses on bacterial synthesis of Indole-3-acetic acid (IAA) for plant growth improvement.

**Methods:**

In the current study, the PGPB were selected on the basis of their auxin production potential and their growth promoting attributes were evaluated. Indole-3-acetic acid producing potential of two selected bacterial isolates was observed by varying different growth conditions i.e., media composition, carbon sources (glucose, sucrose and lactose) and different concentrations of precursor. Influence of various physiological factors (temperature and incubation time period) on IAA production potential was also evaluated.

**Results:**

Both the bacterial strains *Bacillus cereus* (So3II) and *B. subtilis* (Mt3b) showed variable potential for the production of bacterial IAA under different set of growth and environmental conditions. Hence, the IAA production potential of the bacterial isolates can be enhanced by affecting optimum growth conditions for bacterial isolates and can be used for the optimal production of bacterial IAA and its utilization for plant growth improvement can lead to better yield in an eco-friendly manner.

## Introduction

Plant hormones are substantial for plant growth and development this was revealed in the era of Darwin who successfully study behavior, translocation and development of one of the most important phytohormone named Auxin. Auxin was the first plant phytohormone to be discovered from coleoptile tip and actually a key hormone as far as plant growth and development are concerned. There are seven types of phytohormones that directly and indirectly influence plant growth and development and hence very important in plant life. All these phytohormones are naturally produced by plants and also procured from soil and microbiota (Park et al., 2017; Tanimoto, 2005).

Auxin is a heterogeneous group of carboxylic acid signaling molecule responsible for regulating various physiological processes of plants. Auxin is synthesized in aerial parts (shoot apex) and transported to sub-aerial parts of the plants actively by utilizing two common transportational pathways through phloem towards the root via non-directional passive pathway or via cell to by directional active pathway called polar auxin transport (Park et al., 2017). Indole-3-acetic acid (IAA) is a potent signaling molecule essential for plant–microbe interactions and improve plant growth directly (Matsuda et al., 2018). Bacterial auxin changes the auxin pool to either supraoptimal or optimal level and hence improve plant root growth especially the development of secondary roots thereby improving root surface area that promotes plant nutrition and consequently results in better growth and yield of plant. Auxin is synthesized with the help of precursor secreted via the root exudates as per plant genotype. There are three most common pathways involved in biosynthesis of auxin by plant growth-promoting bacteria (PGPB) simultaneously which may be utilized by some bacteria these highlighting that auxin is of prime importance for the plant development. Auxin changes the overall biology of the plants and facilitates other mechanisms of action used by PGPB to trigger plant growth and development (Olanrewaju, Glick & Babalola, 2017).

Most of the rhizobacteria are able to synthesize IAA although some phyllospheric bacteria also produce IAA. This bacterial IAA changes the plant auxin pool to either optimal or supraoptimal levels and improves plant growth directly hence proved to be crucial for better development of plants (Iqbal, Wagi & Ahmed, 2017). Bacterial IAA has phytostimulatory impacts and improves plant growth by improving plant roots development and increasing the surface area to volume ratio of roots and consequently results in better uptake of water and nutrients (Ahmed & Hasnain, 2010). The current study deals with the study of various growth promoting attributes of plant growth-promoting rhizobacteria (PGPR) isolates and impacts of various factors i.e. carbon source, media and tryptophan concentration on the synthesis of bacterial auxin. In addition to this, bacterial IAA was further analyzed through Fourier transform infrared spectroscopic (FTIR) analysis and thin layer chromatographic (TLC) analysis.

## Materials and Methods

### Isolation and screening

A total of ten bacterial strains were isolated from rhizosphere of *Solanum nigrum* (So3I, So3II, So3III, So3IV, So3V So6I, So6II and So6III) and of *Malvastrum tricuspidatum* (Mt3b, Mt6a) following Ilyas et al. (2012). Two isolates were selected on the basis of their auxin production potential following Ahmed & Hasnain (2010) using colorimetric analysis.

### Characterization of bacterial isolates

#### Molecular study

Bacterial strains were identified by extracting the bacterial DNA followed by DNA amplification using universal primers 27F (5′-AGAGTTGATCCTGGCTCAG-3′) and 1492R (5′-CGGCTACCTTGTTACGACTT-3′). The amplified product was sequenced using Automated Sequencer and the obtained sequences were submitted to GenBank for accession numbers. The phylogeny was assessed to study their phylogenetic relationship and the phylogenetic tree was constructed via neighbor-joining method using the sequences obtained by the software MEGA7.

#### Macroscopic study

Bacterial colony morphology was recorded. Various characteristics of bacterial colonies i.e., size, shape, elevation, margin and clarity were observed following Patel & Patel (2014).

#### Microscopic study

Bacterial strains were then subjected to microscopic observation and their shape, size, motility and gram staining were recorded following Cappuccino & Sherman (1992).

#### Physiological study

Both the bacterial strains were characterized physiologically following Ahmed & Hasnain (2010).

### Growth promoting attributes

Growth promoting attributes of both the strains were evaluated by studying direct and indirect mechanisms for plant growth promotion. Different growth promoting attributes studied were IAA production potential, phosphate solubilization, Siderophore production, Cylopropane-1-Carboxylate (ACC) deaminase activity and HCN production potential. Three replicates were used for each treatment in the current study.

#### IAA production potential

Both the isolates were evaluated for their potential to produce bacterial IAA following Ahmed & Hasnain (2010) in the presence and absence of precursor (tryptophan). The data were analyzed statistically using DMR test.

#### Phosphate solubilization

Phosphate solubilization was studied using Pikovskaya Agar plate assay which is an in vitro assay for phosphate solubilization following Paul & Sinha (2015). Phosphate solubilization index and phosphate solubilization efficiency were calculated from inhibition zones using the following formulas:

- PSI = (Colony diameter + Halozone diameter)/colony diameter
- PSE (%) = ((Halozone diameter − Colony diameter)/Colony diameter) * 100

#### Siderophore production

Siderophore production was analyzed quantitatively using CAS shuttle assay following Christina Jenifer et al. (2015).

#### 1-Amino cyclopropane-1-carboxylate deaminase activity

1-Amino cyclopropane-1-carboxylate deaminase activity was analyzed using colorimetric assay following Li et al. (2011).

#### Ammonification potential

Bacterial cultures were tested for the production of ammonia using Nessler’s reagent following Cappuccino & Sherman (1992). Freshly grown cultures were inoculated in peptone broth and incubated for 3 days at 28 ± 2 °C. Nessler’s reagent (0.5 ml) was added in inoculated peptone broth. Development of yellow to brown color was a positive test for ammonia production.

### Study of bacterial auxin production potential

#### Impact of physiological parameters

Impact of temperature, incubation time period and pH was recorded on auxin production potential following Nalini & Tirupati Rao (2014) with slight modifications.

#### Impact of media and growth components

##### Media composition

Two bacterial growth media i.e., Luria Bertani and Yeast Extract Mannitol were used in the current study to optimize the production of bacterial IAA. The data were analyzed statistically using DMR test.

##### Precursor

Auxin production potential of bacterial isolates was recorded under the influence of various levels of precursor i.e., tryptophan concentrations following Nalini & Tirupati Rao (2014).

##### Carbon source

Impact of carbon source (glucose, lactose and sucrose) on bacterial auxin production potential was checked following Nalini & Tirupati Rao (2014). The data were analyzed statistically using DMR test.

##### Wall affecting agents

Impact of wall affecting agents like sodium dodecyl sulfate (SDS) and ethylenediaminetetraacetic acid (EDTA) on bacterial auxin production potential was checked following Nalini & Tirupati Rao (2014). The data were analyzed statistically using DMR test.

### Validation of bacterial auxin production potential

Auxin production potential of the selected bacterial isolates was further confirmed using advanced techniques like TLC and FTIR analysis to confirm auxin producing potential from selected bacterial isolates.

#### Thin layer chromatographic analysis

Thin layer chromatographic analysis was performed following Torres-Rubio et al. (2000) and Rf values of bacterial IAA were compared with standard IAA (Sigma).

### Fourier transform infrared spectroscopic analysis

Fourier transform infrared spectroscopic analysis of bacterial IAA was performed following Patel & Patel (2014) and IR spectra were evaluated and compared with that of standard IAA (Sigma).

## Results

### Isolation and screening

**Figure 1:**
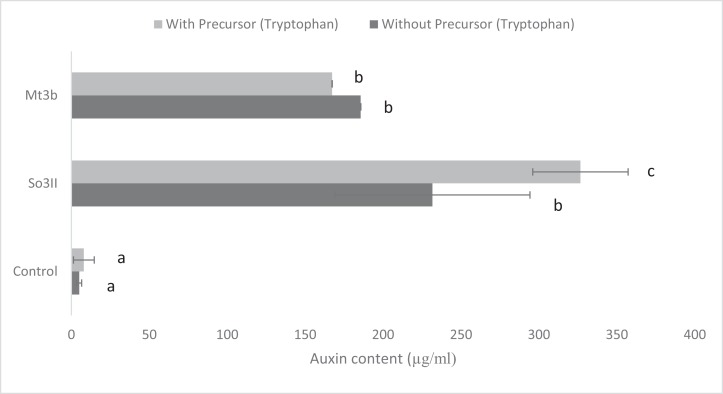
IAA production potential of bacterial isolates in the presence and absence of precursor (Control = IAA synthesis without bacterial inoculation).

In the present study, eight bacterial strains were isolated from *S. nigrum* (So3I, So3II, So3III, So3IV, So3V, So6I, So6II and So6III) and two bacterial strains were isolated from rhizosphere of *M. tricuspidatum* (Mt3b, Mt6a). The auxin production potential of these bacterial isolates was checked and only two bacterial strains i.e., So3II and Mt3b were selected on the basis of their high auxin production potential, both in the presence and absence of precursor i.e., tryptophan. All the bacterial isolates (So3I, So3III, So3IV, So3V, So6I, So6II, So6III and Mt6a) were negative for auxin production potential (Fig. 1).

### Characterization of bacterial isolates

#### Molecular study

**Figure 2:**
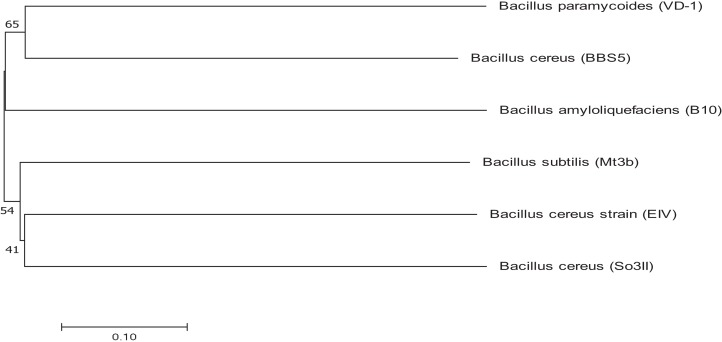
Phylogenetic tree exhibiting phylogenetic relationship between *Bacillus subtilis* (Mt3b) and *Bacillus cereus* (So3II).

Bacterial strains were identified using 16S rRNA sequencing and the obtained sequences were submitted to Genbank for accession numbers. Both the isolated strains were identified as *Bacillus*. The isolated strains were identified as *Bacillus cereus* (So3II) and *B. subtilis* (Mt3b) with accession numbers KM438011.1 and KT025250.1 respectively. *Bacillus cereus* (So3II) and *B. subtilis* (Mt3b) are closely related to each other and have a common ancestor. These strains *B. cereus* (So3II) and *B. subtilis* (Mt3b) have shown bootstrap value of 51 and 41, respectively (Fig. 2).

#### Microscopic study

Both the bacterial strains i.e., *B. cereus* (So3II) and *B. subtilis* (Mt3b) are Gram-positive, motile and spore-forming rods.

#### Macroscopic study

*Bacillus cereus* (So3II) has shown white color, irregular, entire, opaque colonies which are 8–9 mm in size while off white, round, entire, opaque colonies which are 2 mm in size were recorded in *B. subtilis* (Mt3b).

#### Physiological study

Optimum temperature for the growth of these strains *B. cereus* (So3II) and *B. subtilis* (Mt3b) was 37 and 25 °C and optimum pH for their growth was recorded to be seven for *B. cereus* (So3II) and *B. subtilis* (Mt3b). Optimum growth was recorded after 24 and 48 h of incubation time period for *B. cereus* (So3II) and *B. subtilis* (Mt3b), respectively.

### Growth promoting attributes

#### IAA production potential

Both the bacterial isolates *B. cereus* (So3II) and *B. subtilis* (Mt3b) showed high (35.8 and 36.6 µg/ml) auxin production potential in the presence of tryptophan but slightly less concentration of auxin was recorded in the absence of tryptophan precursor i.e., 18 and 20 µg/ml (Fig. 1).

#### Phosphate solubilization

Both the bacterial isolates *B. cereus* (So3II) and *B. subtilis* (Mt3b) have the ability to solubilize inorganic phosphorus and phosphate solubilization index recorded was 1.24 and 1.31 cm respectively and showed phosphate solubilization efficiency was 73% and 78% respectively.

#### Siderophore production

Both the bacterial isolates *B. cereus* (So3II) and *B. subtilis* (Mt3b) were screened on CAS blue medium and both have shown positive results for siderophore production. The percentage of siderophore production recorded was 74.2% and 64.4% in *B. cereus* (So3II) and *B. subtilis* (Mt3b), respectively. While both the isolates produced yellow colonies on CAS Blue agar media.

#### 1-Amino cyclopropane-1-carboxylate deaminase activity

Both isolates synthesize ACC deaminase. 1-Amino cylopropane-1-carboxylate deaminase activity recorded in *B. cereus* (So3II) and *B. subtilis* (Mt3b) was 0.147 and 0.146 mmol/l, respectively.

#### Ammonification potential

Development of yellow to brown color was an indication of ammonification potential. *Bacillus cereus* (So3II) showed the production of ammonia while *B. subtilis* (Mt3b) does not show positive results for ammonification.

### Study of bacterial auxin production potential

#### Impact of physiological parameters

The optimum temperature for IAA production potential of *B. cereus* (So3II) was 37 °C. *B. cereus* (So3II) showed 49.33 µg/ml auxin production potential. *Bacillus subtilis* (Mt3b) has shown IAA production potential of 19.79 µg/ml at 25 °C. Optimum IAA producing potential was recorded after 24 h of incubation time period. This gradually decreases with the passage of time. *Bacillus subtilis* (Mt3b) showed 322.6 µg/ml and *B. cereus* (So3II) has shown 241.6 µg/ml auxin concentrations after 24 h of incubation time period.

#### Impact of media and growth components on auxin production potential

##### Media composition

**Figure 3:**
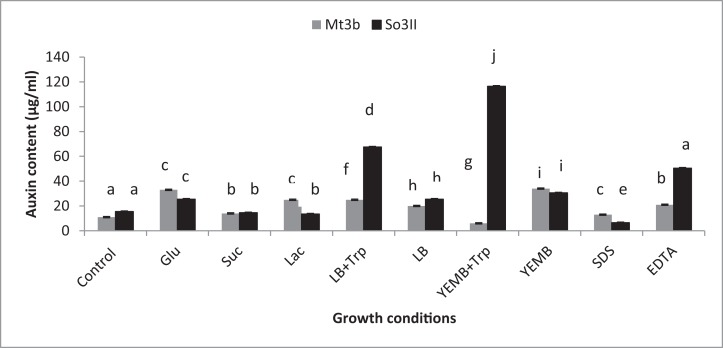
Effect of Carbon source, growth medium, precursor and cell wall affecting substances on biosynthesis of bacterial IAA. Glu, Glucose; Suc, Sucrose; Lac, lactose; LB, Luria–Bertani medium; Trp, Tryptophan; YEMB, Yeast extract mineral broth; SDS, Sodium dodecyl sulfate; EDTA, Ethylenediaminetetraacetic acid. Different letters indicate significant difference between treatments using Duncan’s multiple range test (*P* = 0.05).

Both cultural medium proved to be good for IAA production in LB medium, bacterial isolate *B. subtilis* (Mt3b) showed 27.3% increase in auxin concentration in the presence of tryptophan precursor while 19.6% increase in auxin production was recorded in the absence of tryptophan. Bacterial isolate *B. cereus* (So3II) showed 69% and 22.6% improvement in auxin production potential in the presence and absence of tryptophan precursor, respectively. In yeast extract mannitol broth (YEMB) bacterial strain *B. subtilis* (Mt3b) showed 112% and 31.33% increase in auxin content in the presence and absence of precursor while bacterial isolate *B. cereus* (So3II) improved 13.3% and 32.3% increase in IAA content in the presence and absence of tryptophan respectively, as compared to control (Fig. 3).

##### Precursor

**Figure 4:**
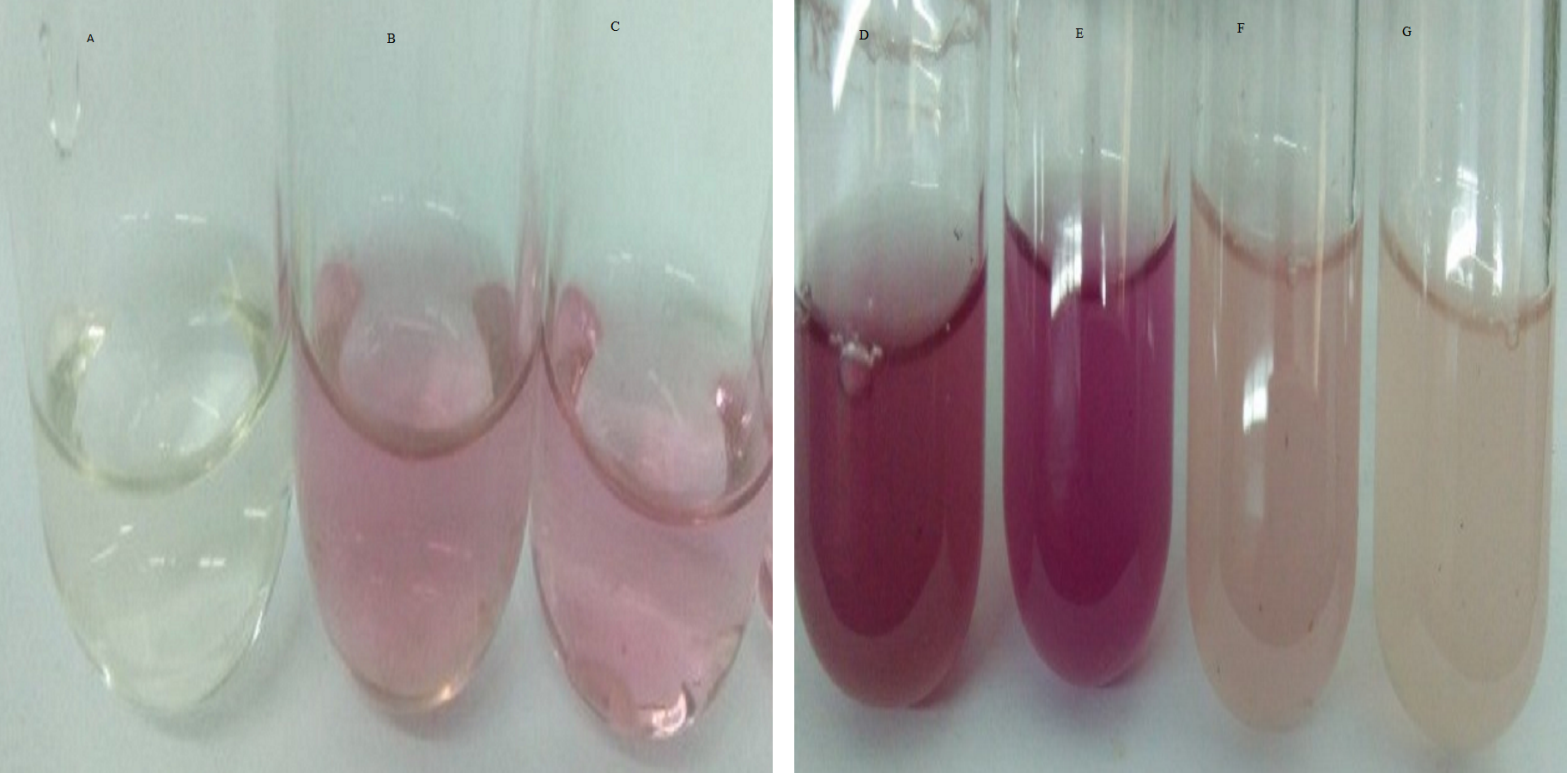
IAA production potential of bacterial isolates. (A—Control; B, C—Effect of precursor; D, E—Effect of EDTA; F, G—Effect of SDS).

Indole-3-acetic acid production potential was recorded at 0–40 mg/ml tryptophan concentration. Both bacterial strains have shown IAA production potential in the presence and absence of tryptophan. Best IAA production was recorded in the presence of 20 and 30 mg/ml of precursor concentration. *Bacillus subtilis* (Mt3b) and *B. cereus* (So3II) showed auxin potential of 37, 54, 44 and 64 µg/ml respectively (Fig. 4).

##### Carbon source

Different bacterial species have the ability to utilize different carbon sources for its growth and interaction with these carbon sources results in the variability in their auxin production capabilities. In the current study basic yeast extract mineral media is supplemented with different carbon sources (1%) i.e., glucose, sucrose and lactose. *Bacillus cereus* (So3II) and *B. subtilis* (Mt3b) use glucose as a carbon source for synthesis of bacterial IAA. These isolates have shown 75% and 68% increment in auxin as compared to control when glucose was supplemented as a carbon source (Fig. 3). *Bacillus cereus* (So3II) and *B. subtilis* (Mt3b) utilize lactose more efficiently as a carbon source to synthesize bacterial IAA and both the isolates have shown 76 and 87% increment in auxin content as compared to control (Fig. 3). *Bacillus cereus* (So3II) and *B. subtilis* (Mt3b) utilize sucrose as carbon source for IAA synthesis and both the isolates have shown 87% and 86% auxin as compared to control (Fig. 3).

##### Wall affecting agents

**Figure 5:**
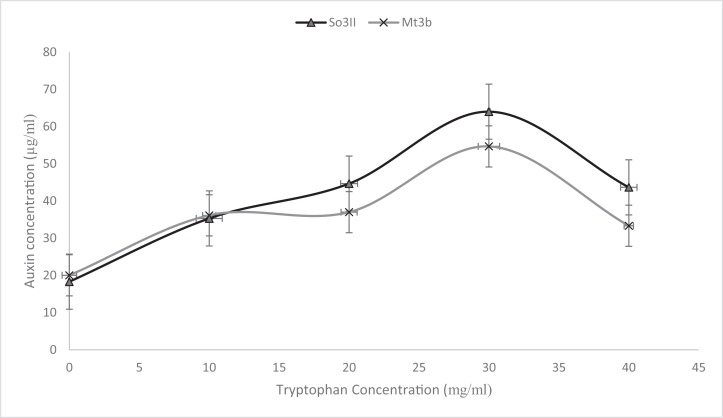
Effect of various concentrations of L-tryptophan on bacterial IAA biosynthesis.

SDS and EDTA both are wall degrading or softening agent that improves IAA release from bacterial isolates. *Bacillus subtilis* (Mt3b) and *B. cereus* (So3II) have shown 80% and 55.6% increment in IAA production potential with SDS while both the strains *B. subtilis* (Mt3b) and *B. cereus* (So3II) have shown 94.6% and 88.3% increase in IAA production potential with EDTA respectively, as compared to control (Figs. 3 and 5).

### Validation of auxin production potential

#### Thin layer chromatographic analysis

Both the isolates *B. cereus* (So3II) and *B. subtilis* (Mt3b) have shown Rf values i.e., 0.951 and 0.952 which were compared with standard IAA (Sigma) having Rf value of 0.956.

#### Fourier transform infrared spectroscopic analysis

**Figure 6:**
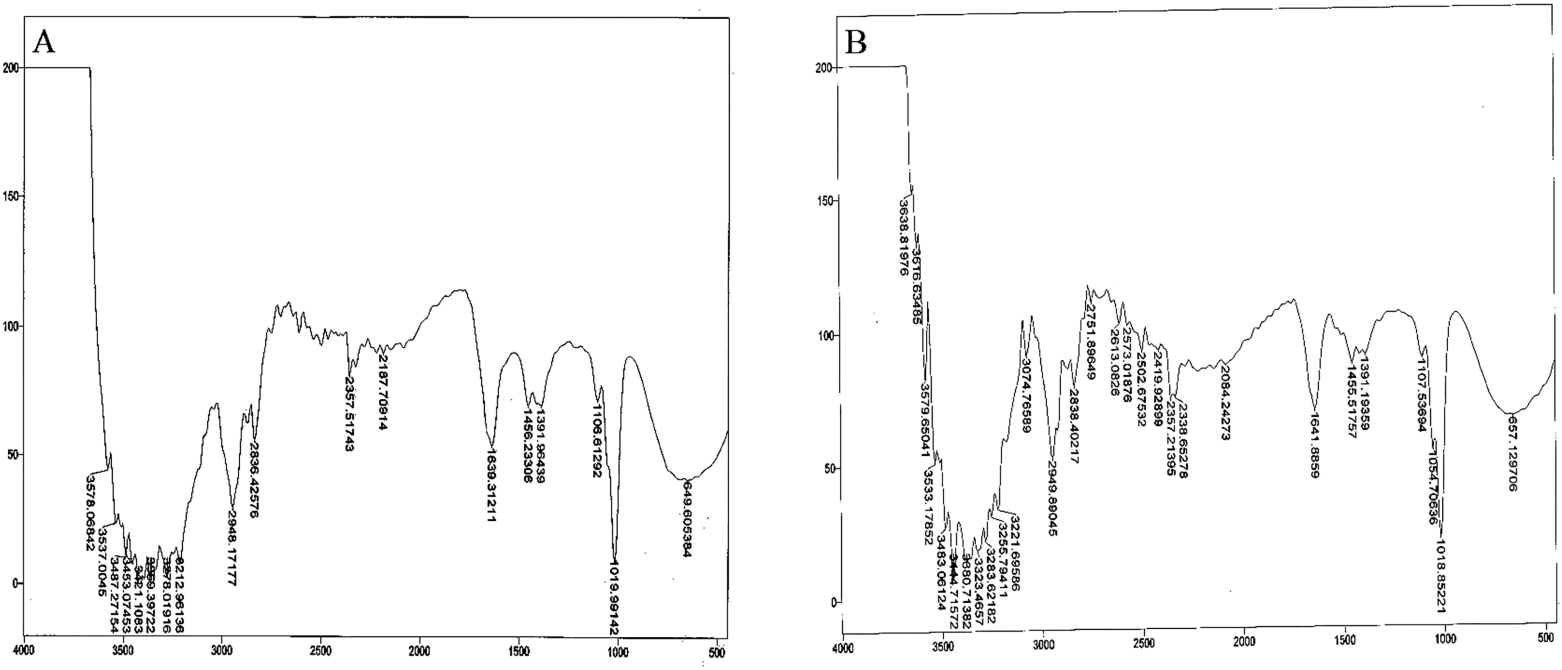
IR spectra of bacterial IAA. (A) Synthetic IAA (B) *B. subtilis*.

Bacterial auxin production was confirmed through FTIR analysis. For the strain So3II and Mt3b, the characteristic peak of OH group appeared in the region of 2,400–3,400 cm^−¹^. The characteristic peak of aromatic ring (C=C) appears in the region of 1,500 cm^−1^ whereas C–N stretch appears in the range of 1,000–13,500 cm^−¹^.The peak of N–H stretch appears in the region of 800 cm^−¹^. All these peaks were very similar to the standard IAA. The characteristic peak for the OH group appeared in the range of 2,400–3,400 cm^−¹^. The characteristic peak of aromatic ring (C=C) appeared in the region of 1,500 cm^−¹^ and CN stretch appears in the range of 1,000–13,500 cm^−¹^ while the peak of N–H stretch was observed in the region of 800 cm^−¹^. Peaks of So3II were most similar to the standard IAA (Sigma) (Fig. 6).

## Discussion

Plant growth-promoting bacteria have a strong potential for plant growth promotion. These plant growth-promoting small entities effectively help in improving plant growth and yield naturally without any side effects. These microbial factories can be utilized to improve plant growth and defend plants from disease-causing pathogens. The current study deals with the evaluation of these PGPB that improve overall plant biology and give us better yield. Two *Bacillus* species were evaluated in the current study and their growth promotional potential was checked. Plant growth-promoting bacteria have the potential to synthesize auxin, which is similar to plant auxin and helps in plant growth and development. Auxin producing PGPR improve plant growth even under stress in the presence of inhibitory compounds by mitigating the effect of all the inhibitory compounds effectively. Both IAA and ACC improve plant growth equally well in stress conditions. Plant roots secrete tryptophan in the rhizosphere which is utilized by rhizobacteria as a precursor for IAA biosynthesis (Shameer & Prasad, 2018).

Ten bacterial strains (So3I, So3II, So3III, So3IV, So3V, So6I, So6II, So6III, Mt3b and Mt6a) were isolated from the rhizosphere of *S. nigrum* and *M. tricuspidatum*. These bacterial strains were screened on the basis of their auxin production potential and only two were selected for further study i.e., Mt3b and So3II. These strains were then subjected to characterization and identified as *B. cereus* (So3II) and *B. subtilis* (Mt3b) (Fig. 1). High auxin production potential was recorded in both the isolates i.e., *B. cereus* (So3II) and *B. subtilis* (Mt3b). Plant growth dramatically improved due to treatment with PGPB. High auxin production was recorded in the presence of precursor i.e., 35.8 and 36.6 µg/ml in the isolates *B. cereus* (So3II) and *B. subtilis* (Mt3b) respectively. Both the strains have shown the ability to produce auxin in the absence of precursor as well (Fig. 2). Tryptophan is an important amino acid secreted by plants as an exudate and bacteria present in the vicinity of these plants develop a mechanism to utilize this amino acid as a precursor and produce plant hormone IAA by utilizing its own biochemical machinery. *Bacillus subtilis* has shown maximum ability for phosphate solubilization as studied by Ahmad et al. (2018). Phosphate solubilizing bacteria improve phosphate availability to plants since in acidic and basic soil, phosphate available is reduced in the plant roots due to strong bonding with calcium and magnesium but bacterial isolates secrete certain enzymes that disrupt this linkage and help to improve the phosphate availability to the plants (Solanki, Kundu & Nehra, 2018). Such bacteria that have high phosphate solubilization efficiency to help plant grow in salt-affected soil. They secrete certain acids that help in maintaining the soil pH and convert phosphate into such forms that become available to the plant roots (Ahmad et al., 2018). *Bacillus subtilis* (Mt3b) produced maximum percentage of siderophore unit i.e., 78%. Microorganisms produce siderophores that help in sequestering iron from the soil and its metabolism efficiently. Iron is the most abundant metal in the earth crust but not readily available to the living organisms because it is not present in soluble forms so siderophore help in the acquisition of iron (Rizzi et al., 2018). *Bacillus cereus* (So3II) and *B. subtilis* (Mt3b) have shown ACC deaminase activity of 0.147 and 0.146 mmol/l, respectively. Bacteria improve plant growth even under stress conditions by the advent of IAA and ACC synergistically (Shameer & Prasad, 2018). Plant growth-promoting bacteria produce ACC deaminase enzyme that regulates production and metabolism of ethylene. Plants overcome various types of salt and metal stress and improve plant growth with the advent of these enzymes. It also helps in the production of deep roots that improve water acquisition (Grobelak et al., 2018). *Bacillus cereus* (So3II) has shown the ability to produce ammonia. The ammonifying bacteria which are involved in the conversion of organic nitrogen to ammonium salt or ammonia, a process named as ammonification, act as natural biofertilizer for plant growth improvement (Zulfarina et al., 2017). Both the isolates *B. cereus* (So3II) and *B. subtilis* (Mt3b) have shown Rf values which are comparable and close to standard IAA. Sardar & Kempken (2018) reported tryptophan dependent IAA production in some microorganisms. Fourier transform infrared spectroscopic analysis was carried out and So3II has shown exact similarity with the standard IAA peaks which confirm that bacteria synthesize IAA (Fig. 6).

Optimum IAA producing potential was recorded after 24 h of incubation and maximum amount of IAA produced was 322.6 µg/ml by *B. subtilis* (Mt3b). Optimum temperature for maximum IAA production potential was 37 °C. Thus, these bacteria produce IAA under mesophilic conditions. Among the various carbon sources used, glucose appeared to be an excellent source since *B. cereus* (So3II) and *B. subtilis* (Mt3b) have shown 75% and 68% auxin synthesis as compared to control. Ethylenediaminetetraacetic acid was an ideal wall softening agent that improved IAA production potential by both *B. subtilis* (Mt3b) and *B. cereus* (So3II) up to 94% and 88% respectively, as compared to control (Figs. 3 and 4). Alamdar, Rasekh & Yazdian (2018) reported increase in growth and biosurfactant production when bacterial strains were treated with SDS nanoparticles at a concentration of 1 mg/ml and have shown no side effect at this concentration. Ethylenediaminetetraacetic acid also improved auxin production potential. According to Kaurin, Cernilogar & Lestan (2018), EDTA application in the calcareous soil is useful for the removal of Pb and also improves soil bacterial diversity and prevent fungal infection. Both cultural medium proved to be good for IAA production. But in case of YEMB medium, amount of auxin synthesized was less. LB medium has shown excellent results both in the presence and absence of tryptophan. YEMB medium was excellent for IAA production in the presence of tryptophan while L-tryptophan is a precursor used in the biosynthesis of bacterial IAA. Plant growth regulator IAA was detected from culture filtrates using TLC technique. Sharma, Sharma & Kaur (2018) also reported the detected bacterial IAA using TLC technique. Bacterial IAA was also analyzed through FTIR analysis which further confirmed the secretion of IAA by the bacteria having the functional groups comparable to those of standard IAA (Fig. 6). Thus, these techniques became very helpful in enhanced production of bacterial IAA which can serve as an efficient plant growth-promoting attribute of PGPR to be considered for effective application of PGPR for growth enhancement.

## Conclusions

The above study suggests that plant growth bacteria can exhibit more than one plant growth-promoting attributes. These attributes interact with each other to synergistically improve plant growth. Bacterial IAA proved to be an efficient metabolite affecting plant growth promotion by the bacterial isolates. Various physiological factors such as presence or absence of precursor, availability of substances including varying media compositions, wall affecting agents and carbon sources affect the synthesis of bacterial IAA. Therefore, they should be optimized to get maximum plant growth improvement when the bacteria are utilized as biofertilizers.

## Supplemental Information

10.7717/peerj.7258/supp-1 Supplemental Information 1 Gene Sequences of the *Bacillus cereus* and *Bacillus subtilis*. 16S rRNA gene sequences of the isolates (*Bacillus cereus* and *Bacillus subtilis*).

10.7717/peerj.7258/supp-2 Supplemental Information 2 Supplementary data file. Data file (Effect of physiological factors, growth curve and growth promoting attributes) of the isolates.

## Additional Information and Declarations

### Data Availability

The following information was supplied regarding data availability: Data are available at GenBank: *Bacillus cereus* (So3II), Accession no. KM438011.1; *Bacillus subtilis* (Mt3b), Accession no. KT025250.1.
