# Supplementary material for: Bacillus spp.: potent microfactories of bacterial IAA
Source: PeerJ. 2019 Jul 23;7:e7258. doi: 10.7717/peerj.7258 (PMC6659656; doi:10.7717/peerj.7258)
Supplement: Supplemental Information 1 — 16S rRNA gene sequences of the isolates (Bacillus cereus and Bacillus subtilis). [file peerj-07-7258-s001.docx]

***Bacillus subtilis***

**Mt3b**

GNNCNGCTTTTCGTAGGTGGCAGCGTTGTCCGGATTATTGGGCGTAAAGGGCTCGCAGGC

GGTTTCTTAAGTCTGATGTGAAAGCCCCCGGCTCAACCGGGGAGGGTCATTGGAAACTGG

GGAACTTGAGTGCAGAAGAGGAGAGTGGAATTCCACGTGTAGCGGTGAAATGCGTAGAGA

TGTGGAGGAACACCAGTGGCGAAGGCGACTCTCTGGTCTGTAACTGACGCTGAGGAGCGA

AAGCGTGGGGAGCGAACAGGATTAGATACCCTGGTAGTCCACGCCGTAAACGATGAGTGC

TAAGTGTTAGGGGGTTTCCGCCCCTTAGTGCTGCAGCTAACGCATTAAGCACTCCGCCTG

GGGAGTACGGTCGCAAGACTGAAACTCAAAGGAATTGACGGGGGCCCGCACAAGCGGTGG

AGCATGTGGTTTAATTCGAAGCAACGCGAAGAACCTTACCAGGTCTTGACATCCTCTGAC

AATCCTAGAGATAGGACGTCCCCTTCGGGGGCAGAGTGACAGGTGGTGCATGGTTGTCGT

CAGCTCGTGTCGTGAGATGTTGGGTTAAGTCCCGCAACGACCGCAACCCGTCATACTGGT

TTTCCTGAGA

***Bacillus cereus***

**So3II**

GGAACGGCTTTATCGTAGGTGGCAGCGTTATCCGGATTATTGGGCGTAAAGCGCGCGCAG

GTGGTTTCTTAAGTCTGATGTGAAAGCCCACGGCTCAACCGTGGAGGGTCATTGGAAACT

GGGAGACTTGAGTGCAGAAGAGGAAAGTGGAATTCCATGTGTAGCGGTGAAATGCGTAGA

GATATGGAGGAACACCAGTGGCGAAGGCGACTTTCTGGTCTGTAACTGACACTGAGGCGC

GAAAGCGTGGGGAGCAAACAGGATTAGATACCCTGGTAGTCCACGCCGTAAACGATGAGT

GCTAAGTGTTAGAGGGTTTCCGCCCTTTAGTGCTGAAGTTAACGCATTAAGCACTCCGCC

TGGGGAGTACGGCCGCAAGGCTGAAACTCAAAGGAATTGACGGGGGCCCGCACAAGCGGT

GGAGCATGTGGTTTAATTCGAAGCAACGCGAAGAACCTTACCAGGTCTTGACATCCTCTG

ACAACCCTAGAGATAGGGCTTCTCCTTCGGGAGCAGAATGACAGGTGGTGCATGGTTGTC

GTCAGCTCGTGTCGTGAGATGTTGGGTTAAGTCCCGCAACGACCGCAACCCGTCATAGTT

GTTTTCCTGATGA
